# An exploratory study on emotion regulation strategy use in individuals with Williams syndrome, autism spectrum disorder and intellectual disability

**DOI:** 10.3389/fpsyt.2022.940872

**Published:** 2022-11-23

**Authors:** Andrea C. Samson, Nayla Sokhn, Jo Van Herwegen, Daniel Dukes

**Affiliations:** ^1^Institute of Special Education, University of Fribourg, Fribourg, Switzerland; ^2^Faculty of Psychology, UniDistance Suisse, Brig, Switzerland; ^3^Department of Psychology, University of Fribourg, Fribourg, Switzerland; ^4^Eye and Brain Mapping Laboratory (iBMLab), Department of Psychology, University of Fribourg, Fribourg, Switzerland; ^5^Department of Psychology and Human Development, UCL Institute of Education, London, United Kingdom; ^6^Swiss Center for Affective Sciences, University of Geneva, Geneva, Switzerland

**Keywords:** emotion regulation, COVID-19, Williams syndrome, autism, anxiety, intellectual disability

## Abstract

**Background:**

Individuals with neurodevelopmental disorders often have atypical emotion profiles, but little is known about how they regulate their emotions. While several studies have examined emotion regulation strategy use in autism spectrum disorder (ASD), only a few have included individuals with intellectual disability (ID) or focused on specific syndromes such as Williams syndrome (WS).

**Methods:**

A parent-reported survey launched during the first months of the COVID-19 pandemic allowed to exploratorily study emotion regulation strategy use and its link to anxiety in individuals with ASD with (N=785) and without ID (N=596), WS (N=261), and Intellectual Disability not otherwise specified (N=649).

**Results:**

Using multilevel analyses, besides revealing specific group differences in emotion regulation strategy use, a variety of strategies (e.g., rumination, avoiding information, repetitive behaviors) were found to be linked to elevated levels of anxiety, while focusing on the positive was linked to lower anxiety levels in all groups. Moreover, only autistic people without ID used humor more frequently while experiencing lower anxiety levels.

**Conclusion:**

This study sheds light on an underexplored area of emotion regulation strategy use in different neurodevelopmental disorders. It also paves the way to further examine emotion regulation in more rigorous ways to better understand emotion regulation in different neurodevelopmental disorders as well as the impact on outcome measures such as anxiety. This exploratory study may help to develop and validate adequate measures to study a broad array of ER strategies used by individuals with neurodevelopmental disorders.

## Introduction

In a variety of contexts, it has been shown that the ability to regulate one's own emotions is important for mental health and wellbeing (1, 2). Emotion regulation (ER) has been defined as the strategies people use to alter the trajectory of an emotion at different points in the emotion generative process to facilitate progress toward a desired goal (2). People use more or less consciously a variety of ER strategies, at times in combination or in sequence, and ideally in a flexible way, to attenuate or itensify their emotional experience. While the context will determine how adaptive the choice and implementation of an ER strategy will be, the adaptiveness of a strategy can also be seen in terms of how often it is used on a habitual basis and its association with long-term outcomes on mental health (2). In this sense, a few strategies are linked to positive long-term outcomes, such as cognitive reappraisal, humor, or problem solving, while others (including avoidance, rumination, or expressive suppression) have been linked to rather negative long-term consequences. Emotion dysregulation, i.e., the inflexible use of more maladaptive ER strategies, has been linked to a variety of internalizing (e.g., anxiety and depression) and externalizing (e.g., aggression) problems (3).

While individuals with neurodevelopmental disorders frequently experience emotional disturbances such as higher internalizing or externalizing problems (4), the link between these problems and ER strategy use has only explicitly been made in a few studies (5, 6). Several studies revealed a tendency for autistic individuals to use maladaptive instead of adaptive ER strategies (7). However, little is known about ER strategy use and efficacy in other neurodevelopmental disorders, such as Williams syndrome (WS), and there are only a few studies about ER strategy use in ASD with intellectual disability (ID) (8). For individuals with ID, more cognitively challenging strategies such as cognitive reappraisal may be less accessible, and the flexibility that is required in different phases of ER (being aware of potential ER strategies, selecting and implementing them appropriately to the context) may be restricted. Furthermore, one needs to constantly monitor ER efficacy in order to maintain, stop or change a specific strategy (2). Limited access to a variety of self-focused ER strategies may render individuals with neurodevelopmental disorders, particularly with ID, more dependent on other people to support their ER, i.e., extrinsic ER (9).

For the purpose of this study, we are interested in comparing the ER strategy use of autistic people with individuals with WS. WS is a rare genetic disorder with an estimated prevalence rate of 1 in 7,500 children (10). In contrast, ASD is relatively more frequent: a recent systematic review indicates a median prevalence of 100 in 10'000 (11). ASD and WS are both neurodevelopmental disorders associated with distinct but overlapping social phenotypes as well as associated psychopathologies, including anxiety. In terms of what distinguishes the social phenotypes, individuals with WS have a socio-emotional profile that can be characterized as being more than usually inclined to approach strangers, being gregarious and hyper-sociable, and being attracted to others' faces (12–15), while the socio-emotional profile of individuals with ASD may be characterized in certain domains as opposite to that (16, 17) with socially-avoidant behavior, a lack of desire to engage with others, and a reduced attention or interest toward social stimuli (15, 17, 18). Interestingly, and in terms of the similarities between the social phenotypes, individuals with WS and autism also share some characteristics, such as difficulties in social cognition and social information processing (e.g., Theory of Mind), social communication, autistic mannerisms and the need to adhere to routines in daily life (16, 17, 19–22).

It is likely that some of these socio-emotional characteristics and difficulties in social cognition and social information processing impact self-focused and extrinsic ER in individuals with neurodevelopmental disorders. Hyper-sociability, positivity bias [which is prominent in WS (23)], and high social approach may, on the one hand, protect individuals from experiencing negative emotions too intensely for too long, but on the other, impact on the availability and choice of ER strategies. Low social motivation and a limited Theory of Mind may render extrinsic ER less accessible to individuals. In addition, further characteristics may impact ER, such as alexithymia [i.e., difficulty to recognize and express emotions and to distinguish between different emotions, as well as thoughts focused on external rather than internal experience (24)], which is frequently present in autistic people (25). Alexithymia may make it difficult to identify the need to regulate, select and implement appropriate strategies or monitor ER efficacy. Finally, the need to adhere to routines may increase the need to regulate. To summarize, several factors may play a role in ER strategy use in individuals with different neurodevelopmental disorders.

In terms of ASD and WS specifically, both are characterized by elevated levels of anxiety (26). Many autistic individuals experience higher trait anxiety (27) and as many as 40% are estimated to meet the diagnostic criteria for anxiety disorder (28). Anxiety is also reported to be one of the most common psychopathologies in individuals with WS (29, 30) with higher levels of anxiety reported than in individuals with Down syndrome, Prader Willi-syndrome, and Intellectual Disability, for example (30–33). Some studies also point to higher anxiety levels in autistic individuals compared to individuals with WS (26, 34). Interestingly, the type of anxieties appears to be different: while autistic people may have social and non-social anxieties, individuals with WS less often show social anxieties compared to autistic people (26).

As already mentioned, while there are some studies concerning ER in ASD, more research is necessary to understand the ER profile in WS. Some studies suggest that individuals with WS may have difficulties with ER affecting anxiety and specific phobias (35–37), but little research has examined ER strategy use in WS. Gaining more insight into ER strategy use in individuals with WS could have important implications for interventions (36).

ER was particularly challenging during the COVID-19 pandemic which presumably almost universally generated high stress and elevated levels of negative emotions. Several studies suggested that maladaptive strategy use such as catastrophizing, rumination, or excessive health-related information seeking was correlated with increased perceived distress, negative emotions, depression, and anxiety, while adaptive strategies (including positive refocusing and acceptance) have acted as a buffer to alleviate emotional distress and negative emotions during COVID-19 in the general population (38–44). It has even been suggested that individuals with neurodevelopmental disorders and their families have been disproportionally impacted (45–47). For example, in a study including individuals with attention deficit and hyperactivity disorder, poorer pre-COVID-19 ER abilities were linked to increased mental health symptoms (48). However, to the best of our knowledge, no study to date has focused on ER strategy use and anxiety in individuals with WS compared to autistic people. A survey launched in the early months of the COVID-19 pandemic presented an opportunity to do just that.

### The present study

The primary goal of this study was to explore the caregiver-reported anxiety and use of a broad array of ER strategies and how the two may be linked in neurodevelopmental disorders. We aimed to include a broad array of ER strategies in an exploratory manner since to the best of our knowledge there is no current validated questionnaire that includes a comparable broad range of behaviors and strategies used in the attempt to deal with anxiety in neurodevelopmental disorders. We were specifically interested in comparing the ER strategy use of individuals with WS and autistic people. Since ID may impact the access to and use of ER strategies, particularly in relation to more cognitively demanding strategies such as cognitive reappraisal, we split the ASD group into individuals with and without ID. Moreover, since it was of interest to study the impact of ID without ASD, we also included individuals with ID not otherwise specified (ID-NOS). First, we compared the level of anxiety in these groups. Second, we described the use of 14 ER strategies representing a variety of adaptive and maladaptive but also rather cognitive and behavioral strategies in these four groups. Twelve strategies were self-focused, while two described co-regulation by parents or caregivers (extrinsic ER). Third, we examined the link between ER strategy use and anxiety across the four groups. Finally, we examined potential group differences in the link between strategy use and anxiety.

## Method

### Participants

For the present study, we will focus on a subsample of the large international sample collected (see procedures section) of 2,288 individuals, in total: 261 individuals with WS (*M* = 17.78 years, *SD* = 10.22, 119 (45.6%) female), 785 autistic people with ID (*M* = 12.42 years, *SD*=7.00, 153 (19.5%) female), 596 autistic people without ID (*M* = 11.49 years, *SD* = 5.92, 124 (20.8%) female), and 649 individuals with ID-NOS (*M* = 19.06 years, *SD* = 12.07, 281 (43.3%) female). The individuals resided in 51 countries (see Supplementary material A). Specific data on race/ethnicity has not been recorded. 65.12% of the respondents were mothers, 27.00% were fathers, and a minority (7.88%) were caregivers or other relatives. The education attainment levels of the respondents were 2.80% with no formal qualification, 13.30% with further vocational training, 20.91% with school-leaving certificate, 35.14% with a university bachelor's degree or equivalent, 21.79 with a university master's degree or equivalent and 6.03% who wrote “Other”.

We found a significant difference in age of the four groups; *F*(3, 2284) = 105.48, *p*<0.001. *Post-hoc* tests revealed that the groups of ASD without and with ID did not differ in age (*p* = 0.22), nor did the groups of WS and ID-NOS (*p* = 0.20). However, autistic people with ID were significantly younger than individuals with ID-NOS (*p* < 0.001, CI = [5.44 7.86]) and individuals with WS (*p* < 0.001, CI = [3.73 7.00]); and autistic people without ID were significantly younger than individuals with ID-NOS (*p* < 0.001, CI = [6.27 8.87]) and those with WS (*p* < 0.001, CI = [4.58 7.99]). The χ^2^-test on distribution of males and females in the four groups revealed a significant effect; χ^2^(3) = 151.98, *p* < 0.001. There were more males than females in each group except in the WS group in which gender was distributed equally (*p* = 0.06). As a consequence, we included age and gender as covariates for the multilevel analyses.

### The survey

The parent or caregiver reported survey was developed at the beginning of the COVID-19 pandemic in order to better understand how individuals with special educational needs and disabilities (SEND; neurodevelopmental disorders constitute a subgroup of SEND) were affected by the pandemic [see (49, 50)]. First, demographic information was requested of the respondent (parents or caregivers) and individual with SEND. The respondent also reported the primary diagnosis and the presence (or absence) of ID in the individual with SEND, allowing us, for example, to distinguish between autistic people with and without ID. Then, anxiety was assessed with a single item [“How anxious was/is your child?”, similar to Turon et al. (51)] on a scale from 1 (not at all) to 5 (extremely) at three time points: before the pandemic, at the start of the pandemic and at the time of survey completion—in the “now” moment (between April and August 2020). Here, we will focus only on the “now” moment since data relating to ER strategy use was only collected about then. Finally, the survey included a broad range of 14 potential ER strategies and asked with single items how frequently from 1 (very rarely) to 5 (very frequently) the individual with SEND used each strategy to deal with the potentially elevated levels of anxiety (the wording of the instructions and ER items can be found in the Supplementary material B, as well as here (49)^1^.

To explore the ER strategy use, we included a range of rather maladaptive and adaptive ER strategies (taking into consideration their habitual use and long-term consequences). Furthermore, we included some cognitive and behavioral strategies, since a broad range of strategies was more likely to capture the relative strengths of each group (2). Twelve of the strategies concerned self-regulation. The following may be considered as rather maladaptive in relation to their long-term consequences: *isolation/withdrawal, information avoidance, information search* [excessively searching for information about the COVID-19 may be an (unsuccessful) attempt to attenuate anxiety levels (41)], *rumination* which refers to continuously thinking about one's own experience, and the causes and consequences of one's negative emotion without calling for action (52), and *expressive suppression*, which refers to inhibiting the outward expression of an emotion (53). Two further possibly maladaptive strategies concerned behavior: *aggressive behaviors* and *repetitive behaviors*. The latter two might not typically be seen as ER strategies but could also be considered as symptoms linked to elevated negative emotions (i.e., anxiety). However, they may also function at times as a response-focused ER strategy. Moreover, repetitive behaviors have been reported to be used as an attempt to deal with negative emotions in several studies (45, 54). In this light, it is important to note that the survey asked which strategies were shown in response to deal with elevated levels of anxiety and stress and not if an individual shows these behaviors in general.

Some strategies may be considered as either adaptive or maladaptive depending on the context such as *sharing/talking about* COVID-19 and *distraction* as one of the attentional deployment strategies (i.e., shifting one's attention to something else in order to avoid or reduce unwanted emotions) (1). Finally, some strategies can be seen as rather adaptive considering their habitual use and long-term consequences: *cognitive reappraisal* (53), *focusing on the positive* (55), and *humor* (56, 57).

Furthermore, we asked about two strategies that we imagined may have been employed by parents or caregivers to regulate the child's emotion: shielding the child from negative information about the pandemic (*parent shielding*) and establishing a routine in their daily life (*parent routine*)^2^.

### Procedures

The survey, developed at the beginning of the COVID-19 pandemic, was available in 16 languages (49, 50). With the help of more than 60 international collaborators, flyers were sent to associations and cohorts to invite parents and caregivers to report about their child with SEND. There was no age limit for the child with SEND, so respondents were also able to report about their adult child. Ethical approval for this anonymous survey was obtained by the institutional review board of Unidistance Suisse.

Due to the necessity of rapidly developing our COVID-19 specific questionnaire in March 2020, we did not include community members or people with SEND in the development of the questionnaire. However, we had several parent associations help us recruit and we are continuing to work with them with regards to the dissemination of the survey results.

### Data analysis plan

We included data of families based on the following selection criteria, resulting in the above-mentioned *N* = 2,288: primary diagnosis of WS, ASD, or ID-NOS, available information about country, age, gender, available information about presence of ID (which allowed us to distinguish between autistic people with and without ID), age 5 and older (since self-focused cognitive strategies are less likely to occur in younger participants). We excluded cases in which the respondents provided inconsistent information (e.g., primary diagnosis ID-NOS, but a negative response when asked if their child had ID). We did not impute missing values for the ER strategy use; instead, we opted for the complete-cases-analysis approach: a participant is removed if an answer is missing for any of the 12 ER strategies.

We performed linear mixed models to investigate (1) the level of anxiety in the different groups, (2) ER strategy use in the different groups, (3) ER profiles linked to anxiety across the four groups, and (4) group differences in the link between ER strategies and anxiety. The independent variable group for all models except for the third is coded as a categorical variable with four modalities (ASD without ID, ASD with ID, ID-NOS, and WS), the reference level is the ASD without ID. In all four models, age and gender were added as covariates and the country was used as a random factor to account for dependency within each country. Finally, all models are estimated using the restricted maximum likelihood (REML). Preprocessing of the data was conducted using MATLAB software (58) (R2018b, The MathWorks, Natick, MA). Analyses were conducted using R statistical software version 4.0.3 (59), with the packages *lme4* and *lmerTest* for multilevel regression (60, 61), *emmeans* (62) for pairwise comparisons using the tukey method for p-value correction, *effects* (63) and *ggplot 2* (64) *packages* for visualization of main effects of the model.

## Results

### Level of anxiety in the different groups

**Figure 1:**
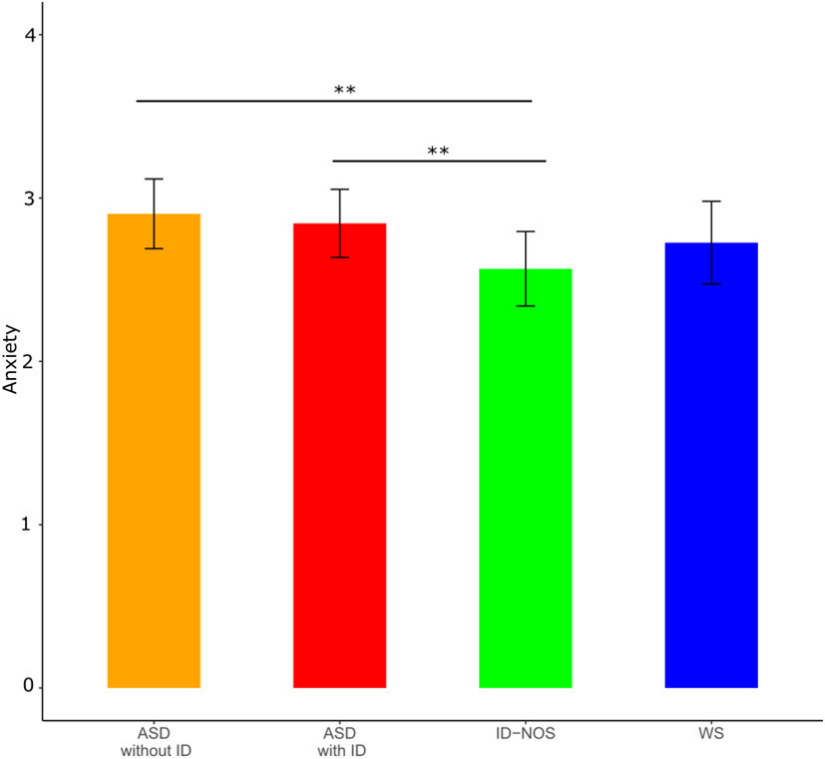
Mean anxiety levels with confidence intervals for each group. Significant effects of *post hoc* tests are indicated (black lines). ASD, autism spectrum disorder; ID, intellectual disability; ID-NOS, intellectual disability not otherwise specified; WS, Williams syndrome. Significance levels of *post-hoc* tests: ^**^p < 0.01.

The analyses of anxiety levels revealed a main effect of group; *F*(3, 2229) = 5.48, *p* < 0.001 and age; *F*(1, 2240) = 33.29, *p* < 0.001, indicating increased anxiety with increasing age, but no effect for gender; *F*(1, 2226) = 0.02, *p* > 0.05. *Post-hoc* tests revealed significant differences between individuals with ID-NOS and autistic people without ID (*t* = 3.68, *p* < 0.01) and autistic people with ID (*t* = 3.46, *p* < 0.01), respectively, showing that autistic people with and without ID had higher levels of anxiety than individuals with ID-NOS (see Figure 1).

### Emotion regulation strategy use in the different groups

On a descriptive level, when sorting the employed ER strategies for each group according to their frequency (see Supplementary material C), it can be observed that *parent routine* and *parent shielding* were among the most frequently parent-reported strategies in all groups. Distraction, repetitive behaviors, and isolation/withdrawal were also frequently employed.

**Figure 2:**
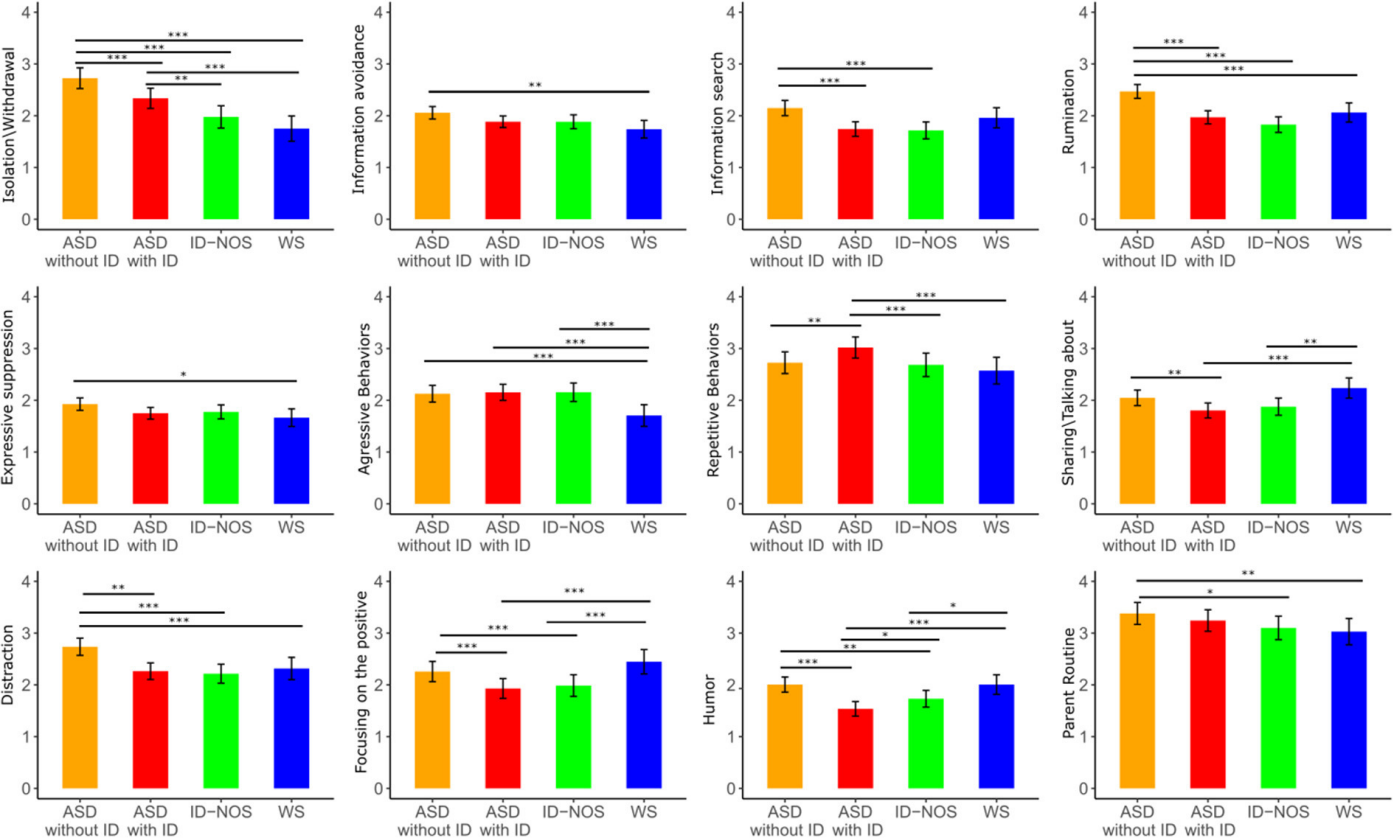
Group differences were found in the use of 12 of the 14 emotion regulation (ER) strategies (frequency: from 1 = very rarely to 5 = very frequently). Mean levels with confidence intervals are shown per group for each ER strategy. Significant effects of *post hoc* tests are indicated (black lines). ASD, autism spectrum disorder; ID, intellectual disability; ID-NOS, intellectual disability not otherwise specified; WS, Williams syndrome; Significance levels of *post-hoc* tests: * p < 0.05, ** p < 0.01, *** p < 0.001.

The analyses of parent-reported use of ER strategies revealed significant group effects in 12 out of 14 ER strategies (see Figure 2). No significant group differences were found for *parent shielding* and *cognitive reappraisal*. Generally speaking, no gender effects were found but there was almost always an age effect indicating that the use of a specific ER strategy increased with increasing age except for *repetitive behaviors, parent shielding*, and *parent routine*, where no age effects were found. This suggests that extrinsic ER and repetitive behaviors do not seem to change in our sample as much as other strategies examined here. For the statistics of group, age, and gender effects (see Table 1). In the following, we focus on *post-hoc* tests within the 12 ER strategies in which significant group effects were found.

**Table 1:** Effects of group, age, and gender within the multilevel analyses on ER strategy use.

**ER strategies**	**Effect of group**	**Effect of age**	**Effect of gender**
Isolation/withdrawal	*F*(3, 2251) = 34.53^***^	*F*(1, 2280) = 27.15^***^	*F*(1, 2267) = 0.00
Information avoidance	*F*(3, 1584) = 4.11^***^	*F*(1, 1997) = 14.86^***^	*F*(1, 1983) = 0.27
Information search	*F*(3, 2062) = 12.44^***^	*F*(1, 2216) = 35.53^***^	*F*(1, 2277) = 1.00
Rumination	*F*(3, 1681) = 22.87^***^	*F*(1, 2038) = 35.05^***^	*F*(1, 2281) = 2.55
Expressive suppression	*F*(3, 1536) = 3.28^*^	*F*(1, 1966) = 16.93^***^	*F*(1, 2282) = 0.00
Aggressive behaviors	*F*(3, 2147) = 7.04^***^	*F*(1, 2249) = 7.95^***^	*F*(1, 2276) = 0.17
Repetitive behaviors	*F*(3, 2245) = 8.43^***^	*F*(1, 2279) = 0.40	*F*(1, 2272) = 0.09
Sharing/talking about COVID-19	*F*(3, 2136) = 8.33^***^	*F*(1, 2244) = 15.16^***^	*F*(1, 2276) = 0.18
Distraction	*F*(3, 2127) = 15.07^***^	*F*(1, 2244) = 11.29^***^	*F*(1, 2275) = 0.40
Cognitive reappraisal	*F*(3, 2026) = 0.97	*F*(1, 2206) = 10.84^**^	*F*(1, 2275) = 0.00
Focusing on the positive	*F*(3, 2248) = 12.38^***^	*F*(1, 2282) = 8.78^**^	*F*(1, 2258) = 1.35
Humor	*F*(3, 2123) = 18.27^***^	*F*(1, 2242) = 6.56^*^	*F*(1, 2275) = 1.80
Parent shielding	*F*(3, 2278) = 0.72	*F*(1, 2277) = 1.45	*F*(1, 2264) = 1.41
Parent routine	*F*(3, 2268) = 4.70^**^	*F*(1, 2282) = 0.20	*F*(1, 2270) = 0.09

ER = emotion regulation.

* p < 0.05,

** p < 0.01,

*** p < 0.001.

#### Isolation/withdrawal

We found significant differences between all the groups except between ID-NOS and WS (see Supplementary material D for *t*-values) with the highest scores for autistic people without ID, followed by autistic people with ID, and the lowest levels for individuals with ID-NOS and WS.

#### Information avoidance

Autistic people without ID more frequently avoided information compared to individuals with WS (*t* = 3.22, *p* < 0.01).

#### Information search

Autistic people without ID search for more information compared to autistic people with ID (*t* = 5.54, *p* < 0.001) and individuals with ID-NOS (*t* = 5.02, *p* < 0.001).

#### Rumination

Autistic people without ID ruminate more frequently than all other groups (compared to autistic people with ID: *t* = −6.77, *p* < 0.001, to individuals with ID-NOS: *t* = −7.42, *p* < 0.001, and to individuals with WS; *t* = −3.92, *p* < 0.001).

#### Expressive suppression

Autistic people without ID used this ER strategy more frequently than individuals with WS (*t* = 2.67, *p* < 0.05).

#### Aggressive behaviors

Individuals with WS used *aggressive behaviors* less frequently than autistic people with ID (*t* = 4.31, *p* < 0.001), autistic people without ID (*t* = 3.93, *p* < 0.001) and individuals with ID-NOS (*t* = 4.07, *p* < 0.001).

#### Repetitive behaviors

Autistic people with ID used *repetitive behaviors* significantly more frequently than all other groups (compared to autistic people without ID: *t* = −3.43, *p* < 0.01, to individuals with ID-NOS: *t* = 3.80, *p* < 0.001, and to individuals with WS: *t* = 3.82, *p* < 0.001).

#### Sharing/talking about COVID-19

Individuals with WS used this ER strategy significantly more frequently than autistic people with ID (*t* = −4.43, *p* < 0.001) and individuals with ID-NOS (*t* = −3.46, *p* < 0.01). Moreover, autistic people without ID used this ER strategy more frequently than autistic people with ID (*t* = 3.41, *p* < 0.01).

#### Distraction

Autistic people without ID used *distraction* more frequently than the other three groups (compared to autistic people with ID: *t* = 6.02, *p* < 0.001, to individuals with ID-NOS: *t* = 5.65, *p* < 0.001, and to individuals with WS: *t* = 3.82, *p* < 0.01).

#### Focusing on the positive

Turning to the rather adaptive ER strategies, a significant group effect was found for *focusing on the positive*. While autistic people without ID and WS did not differ, both groups used this strategy more frequently than the other two groups: Autistic people without ID used *focusing on the positive* more frequently than autistic people with ID (*t* = 4.42, *p* < 0.001) and individuals with ID-NOS (*t* = 3.11, *p* < 0.001). In addition, individuals with WS used this strategy more frequently than autistic people with ID (*t* = −5.09, *p* < 0.001) and individuals with ID-NOS (*t* = −4.27, *p* < 0.001).

#### Humor

Autistic people without ID and WS did not differ, but both used *humor* as ER strategy more frequently compared to the other groups. Autistic individuals with ID seemed to be using this strategy less often than all other groups (autistic people without ID used humor significantly more frequently than autistic people with ID: *t* = 6.78, *p* < 0.001, and individuals with ID-NOS: *t* = 3.36, *p* < 0.01. Autistic people with ID used this ER strategy less frequently than individuals with ID-NOS: *t* =−2.74, *p* < 0.05, and then individuals with WS: *t* =−4.96, *p* < 0.001. Individuals with ID-NOS used this ER strategy less frequently than individuals with WS: *t* = −2.72, *p* < 0.05).

#### Parent routine

Finally, a significant group effect was found for *parent routine*. Parents and caregivers of autistic people without ID used this strategy more frequently than the parents of individuals with ID-NOS (*t* = 3.00, *p* < 0.05) and individuals with WS (*t* = 3.16, *p* < 0.01).

### Emotion regulation strategy use linked to anxiety across the four groups

**Figure 3:**
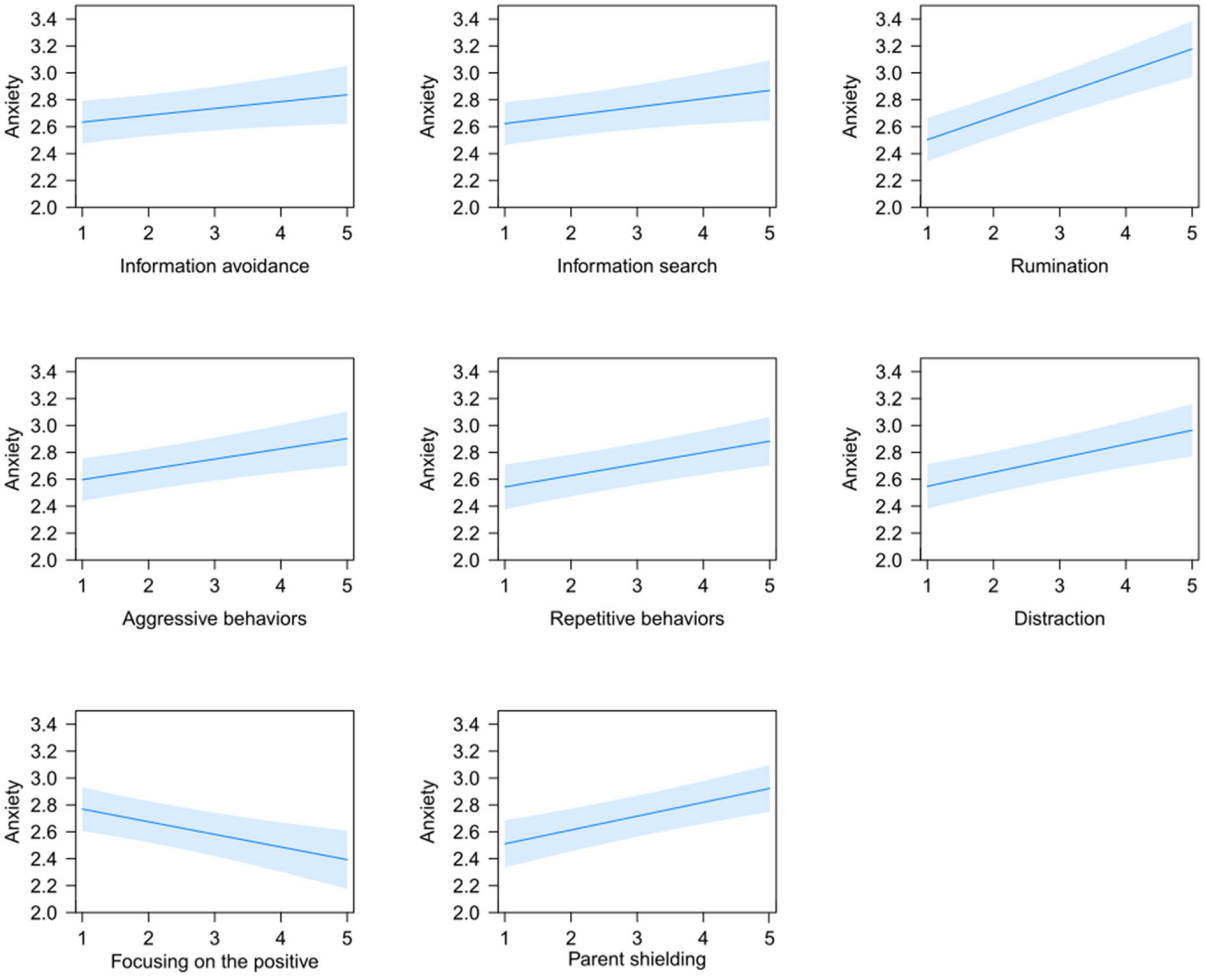
Illustration of the significant association between the frequency (from 1 = very rarely to 5 = very frequently) of emotion regulation strategy use and anxiety levels across the four groups. Blue bands denote the 95% confidence interval.

The analyses of ER strategies across the four groups revealed significant effects for seven strategies that were linked to higher anxiety: *information avoidance*; *F*(1, 2217) = 4.40, *p* < 0.05, *information search; F*(1,2224) = 5.50, *p* < 0.05, *distraction*; *F*(1, 2225) = 20.24, *p* < 0.001, *rumination*; *F*(1, 2223) = 47.60, *p* < 0.001, *aggressive behaviors*; *F*(1, 2221) = 12.31, *p* < 0.001, *repetitive behaviors*; *F*(1, 2224) = 17.45, *p* < 0.001, and *parent shielding*; *F*(1, 2150) = 22.97, *p* < 0.001. Only one strategy, i.e., *focusing on the positive* was linked to lower anxiety; *F*(1, 2227) = 12.69, *p* < 0.001 (see Figure 3). A significant effect was found for age; *F*(1, 2227) = 14.77, *p* < 0.001, indicating a stronger association between strategy use and anxiety with increasing age, but no gender effect was found; *F*(1, 2221) = 0.01, *p*>0.05.

### Group differences in the link between emotion regulation and anxiety

**Figure 4:**
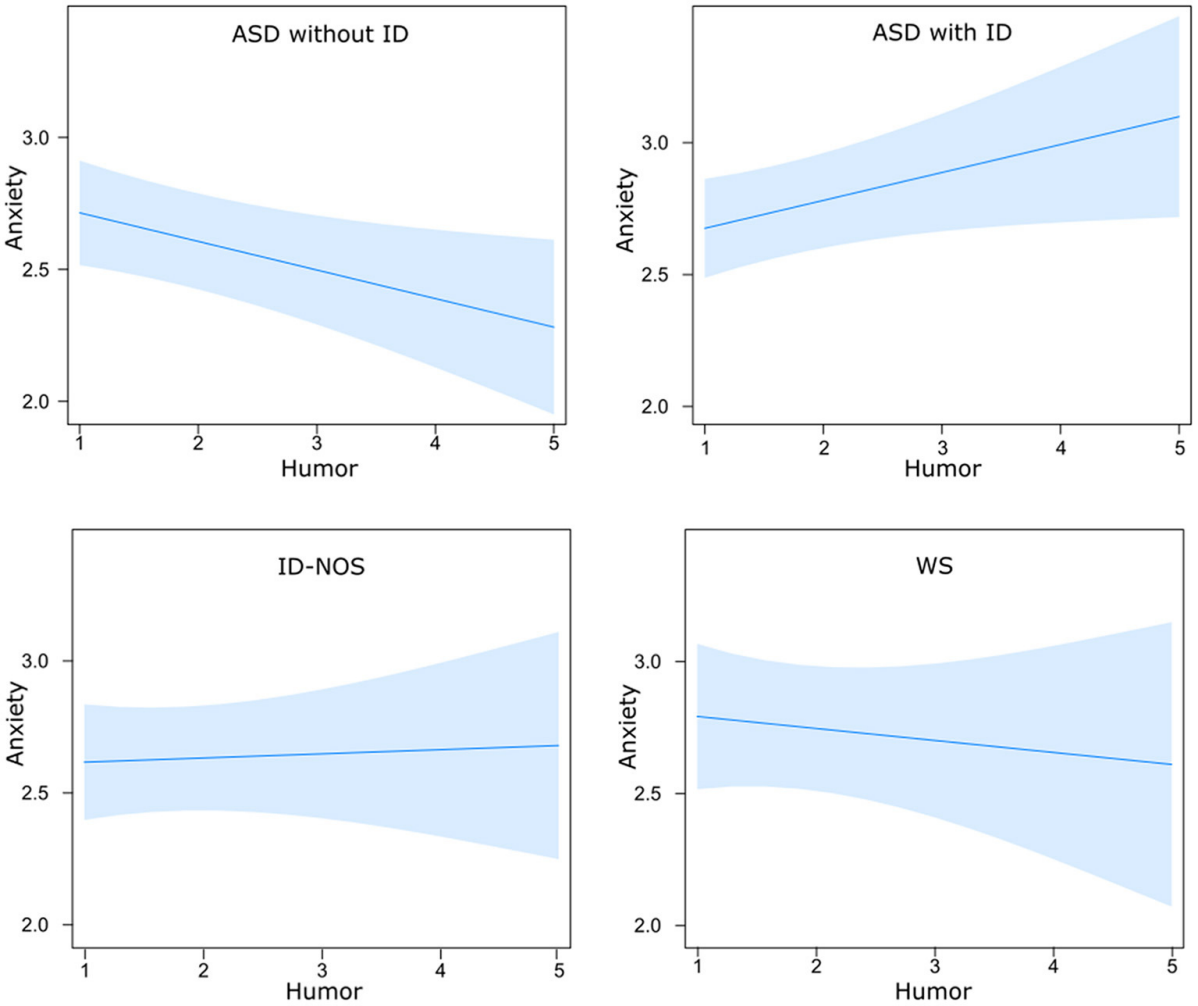
Differential effects of humor on anxiety in relation to group membership: Using humor as an emotion regulation strategy was linked to lower anxiety in autistic individuals without ID and to higher anxiety in autistic individuals with ID. ASD, autism spectrum disorder; ID, intellectual disability; ID-NOS, intellectual disability not otherwise specified; WS, Williams syndrome. Blue bands denote the 95% confidence interval.

We found significant effects of age and of the same seven ER strategies as presented in section Emotion regulation strategy use linked to anxiety across the four groups but not for gender or group (see Supplementary material E for the statistics). However, most interestingly, we found a significant interaction between humor and group; *F*(3, 2163) = 3.67, *p* < 0.05. *Post-hoc* comparisons revealed a significant difference between autistic people without ID and with ID; *t* = - 3.27*, p* < 0.01. This may suggest that only autistic people without ID used humor more frequently while experiencing lower levels of anxiety, since *humor* was linked to lower anxiety in this group. While we did not find an association between humor and anxiety in individuals with WS and ID-NOS, *humor* was more frequently used while experiencing increased levels of anxiety in autistic people with ID (see Figure 4).

## Discussion

Our survey, launched in the first months of the pandemic, allowed us to study ER strategy use and the link to anxiety in neurodevelopmental disorders, namely, in individuals with WS, autistic people with and without ID, and individuals with ID-NOS. Individuals with neurodevelopmental disorders have been disproportionally affected by the COVID-19 pandemic with increased levels of anxiety and other mental health outcomes (46, 47, 65, 66). While our current analysis does not allow a comparison with typically developing individuals, we observed that autistic people (with and without ID) had elevated anxiety levels compared to individuals with ID-NOS, in line with previous studies reporting prevalence rates of 42–79% in autistic people (67) while prevalence rates seem lower in individuals with ID [3–22%, (68)]. While individuals with WS experienced increased anxiety in previous studies when compared to people with ID (30), their anxiety levels in the present study were reported to be between the other three groups. Our study mainly aimed to increase our knowledge about parent-reported ER strategy use and how ER strategy use was linked to anxiety.

It seems striking that *parent routine* and *parent shielding* as extrinsic ER were among the top three most frequently employed strategies in all groups, which shows that co-regulation of negative emotions seems to play an important role in all groups. Further studies may help to better understand if other, more cognitive extrinsic ER strategies, such as *other-employed cognitive reappraisal*, are of greater relevance to individuals with neurodevelopmental disorders than to individuals without. It is also interesting that *repetitive behaviors* are among the four most frequently used strategies in all groups, except for autistic people without ID.

### Emotion regulation profiles in the different groups

Age effects were found for most of the ER strategies indicating increased use of ER strategies as individuals get older. Although we did not find specific age effects for the different groups in relation to ER strategy use, we would like to mention that the use of adaptive or maladaptive ER strategies may be considered as potential protective or risk factors for socio-emotional outcome measures in individuals with neurodevelopmental disorders across the life span. For example, while children and adolescents with WS are often described as overfriendly and of portraying high approach behavior, adults with WS are often described as socially isolated [see, for example Davies et al. (69)]. It may be important to further study the use of ER strategies in different age groups in relation to various outcome measures including anxiety or social isolation in individuals with neurodevelopmental disorders.

Importantly, we identified differences in the ER profiles of the groups. As far as we are aware, this is the first time that the strategy use of individuals with WS has been studied. At a descriptive level, individuals with WS used most frequently *parent routine, parent shielding, repetitive behaviors* and *distraction*, suggesting that extrinsic ER, as well as behavioral ER strategies, are of great relevance. We would like to highlight some characteristics in ER potentially linked to the socio-emotional profile in this rare genetic disorder. In comparison with the other groups with ID, individuals with WS used *focusing on the positive* significantly more frequently and *aggressive behaviors* significantly less. This might be linked to their socio-emotional profile such as the positivity bias in individuals with WS who are often described as gregarious and cheerful (70). In addition, individuals with WS seemed to use humor as frequently as autistic people without ID—which may be again linked to their positivity bias. However, the use of humor was not linked to lower levels of anxiety in individuals with WS (in contrast to the effects of using humor on anxiety in autistic individuals without ID) which may be possibly linked to the particular cognitive challenges related to humor appreciation and production (71). Participants with WS also reportedly used the strategy *sharing/talking about COVID-19* more frequently than the other groups with ID, and the lowest levels of *isolation/withdrawal*, as well as less *information avoidance* and *expressive suppression* than autistic people without ID, which may be linked to the pro-social nature widely reported in WS, characterized by high social interest, social approach behavior and high sociability (70).

Autistic people without ID seemed to have a different ER pattern. Compared to the other groups, autistic people without ID had the highest scores in *isolation/withdrawal* and seemed to *avoid information* more than other groups which seems to be in line with reports about their socio-emotional profile (15, 16). They also seemed to use several cognitive strategies more frequently than the other groups which are all characterized by cognitive impairment: *Search for more information about COVID-19*, which may be linked to the detail-oriented cognitive style in autistic individuals (72), and *rumination*. Interestingly, *focusing on the positive* and *humor* were as frequently employed as by individuals with WS. We may conclude that autistic people without ID are more likely to use and potentially benefit from cognitive strategies (which are potentially too challenging for individuals with ID) because of their typical or better than typical cognitive functioning levels. In addition, establishing a routine by parents and caregivers seems to be highly relevant for autistic people without ID (but also for autistic people with ID, as there were no significant differences between these two groups). This fits well to the known elevated need for routines of autistic people (73).

Our study suggests that autistic people with ID and individuals with ID-NOS have a more limited ER repertoire than the other two groups, by not frequently engaging in cognitive strategies. Despite the limited research on ER in ID [see McClure et al., (74)], this aligns with previous observations of a limited repertoire of coping and ER strategies in individuals with ID (75, 76). In the present study, *Repetitive behaviors* were used most frequently by autistic people with ID compared to the other groups. In the absence of a broader range of (cognitive) strategies available to deal with elevated levels of anxiety, it may be possible that extrinsic ER is more important for autistic people with ID and individuals with ID-NOS. While our study only asked for two extrinsic ER strategies, future studies should include a broader range (e.g., extrinsic cognitive reappraisal, distraction) to better understand which strategies employed by parents or caregivers are used most frequently and are most efficacious. Future research is needed, not only to more rigorously examine self-reported and observed ER strategy use linked to ID and cognitive flexibility, but also to examine, at a more fine-grained scale, in which stages of the ER cycle [identification, strategy selection, strategy implementation, and monitoring (2)] ER might be most affected in individuals with ID. To our knowledge, no research has attempted to conceptualize and tease apart these different stages within the ER cycle with the link to ID (2). While our study focused on the parent-reported implementation of ER strategies, the other stages linked to self- and other-focused ER need to be better understood.

Besides cognitive functioning levels, positivity bias, social approach behaviors/sociability, and the need to adhere to routines, as discussed above, alexithymia and theory of mind are potential factors that may impact ER (see Introduction). With the current study design, we are not able to determine the extent to which these factors impact ER in our target groups. However, future studies assessing these abilities in neurodevelopmental disorders may further elucidate their differential impact on ER.

### Emotion regulation strategy use and its link to anxiety

Several of the ER strategies included in this study were linked to increased anxiety. Many of these (*information avoidance, information search, rumination, aggressive behaviors*) could be considered as rather maladaptive when a strategy is used frequently on a habitual basis, while *distraction* might be adaptive or maladaptive depending on the context (77). *Repetitive behaviors* may actually have an important soothing effect for individuals with neurodevelopmental disorders, particularly if other (cognitive) strategies are less available or accessible (45, 54), even though it may have negative long-term consequences. In general, the literature has shown that if certain strategies are used too frequently, there may have negative long-term consequences on mental health (3). Interestingly, we cannot determine whether these strategies had necessarily a negative effect on emotions, despite their link to increased anxiety levels. It may also be possible that higher anxiety *triggered* the use of a broad range of strategies, including strategies that are considered as rather maladaptive. Also, we need to be clear that we did not ask what the people do on a habitual basis (we rather asked, “which strategies does your child use these days to cope with potentially elevated levels of anxiety”) and we did not employ a longitudinal research design which would allow us to draw conclusions on mid- or long-term consequences. Therefore, the increased use of these strategies may not only reflect unsuccessful attempts to attenuate increased anxiety levels, but they may also be elicited by higher anxiety, suggesting that the increased use of these strategies may also reflect an increased *need* to regulate. Also, our study assessed ER strategies at one time point only. Future studies targeting individuals with different neurodevelopmental disorders should attempt to monitor changes in ER strategy use related to varying levels of anxiety and stress to be able to draw conclusions about potential changes and adaptations of ER strategy selection and implementation. Such studies could be done using ecological momentary assessments (78) if they have been adapted for individuals with neurodevelopmental disorders with and without ID.

Interestingly, only one strategy was more frequently employed when experiencing lower levels of anxiety: *Focusing on the positive*. Even if this might suggest that this could be a powerful strategy to alleviate anxiety in individuals with neurodevelopmental disorders, in line with findings in individuals with typical development (55), we would like to reiterate that we cannot draw conclusions about a causal link in the present study. Future studies should try to shed more light on the question of whether only people with low anxiety levels are actually able to *focus on the positive*, in the face of difficult life circumstances, or if this strategy actually reduces anxiety in neurodevelopmental disorders. This could prove very important. Nevertheless, several intervention-based studies were published in recent years that successfully implemented positive elements such as play and humor to address fear and anxieties in children with WS (79) or that were able to increase the use of positive emotion regulation strategies including *Focusing on the Positive* in individuals with ASD (80).

### Humor as an emotion regulation strategy

While previous studies have shown that humor can be a powerful strategy to regulate emotions [see Samson and Gross (57) for an overview], we found that the use of *humor* as an ER strategy was only linked to lower anxiety in autistic people without ID, suggesting that they may potentially benefit from this strategy, while *humor* was linked to increased anxiety in autistic people with ID. However, again, we need to be cautious in drawing conclusions about a causal link. As shown in a series of studies, autistic individuals have no difficulty to appreciate simpler forms of humor but have more difficulties in understanding and appreciating more complex jokes that require, for example, a Theory of mind to be understood (jokes based on false beliefs) (81). Moreover, lower cheerfulness as a trait and higher seriousness seemed to dampen the susceptibility to humor and humor appreciation in autistic individuals without ID [Samson (82) for a review]. The present study suggests there might be important differences between autistic people with and without ID in terms of their use of humor. It may be possible that producing humor to regulate emotions might have been too overwhelming for autistic people with ID, while it could serve as a resource for autistic people without ID (71). However, this study also opens up room for future research such as examining the difference between producing (which may be more challenging) and consuming humor (for example, watching funny movies) to regulate emotions. Further research could also examine the extent to which autistic people with ID may benefit from humor as extrinsic ER.

### Limitations and future implications

Our exploratory study with a large sample of individuals with different neurodevelopmental disorders included autistic people with ID who are often underrepresented in studies on ER in ASD and, for the first time in such a study of ER strategies, individuals with WS. Future research should ideally attempt to replicate our findings with validated measures (for example, by first developing adequate measures that allow the assessment of a variety of ER strategies in neurodevelopmental disorders) in better characterized samples, and focus on specific ER strategies in more detail in experimental settings. These steps would help formulate concrete implications for application in daily life or interventional settings.

There are some limitations to this study that need to be mentioned. With a view to including a broad range of ER strategies but keeping an already long survey manageable for the families, we assessed ER strategy use with single items rather than including existing ER questionnaires. This helped to gain a better understanding of ER strategy use in neurodevelopmental disorders since, to the best of our knowledge, no existing questionnaire includes such a wide range of strategies. Future studies should therefore develop this battery further for the study of ER in individuals with neurodevelopmental disorders. Moreover, as our motivation was to access as many families with a child with SEND as possible and within a short time frame to cover the early effects of the COVID-19 pandemic, we were neither able to include individuals with SEND in the research process, nor to verify the primary diagnosis, relying instead on parent and caregiver reports. Given that not everyone may have had access to clinics or professionals using gold standard measures for diagnosis [e.g., the Autism Diagnostic Observation Schedule, second edition; ADOS-2; Lord et al. (83) for ASD], this remains a weakness of the study. That said, we hope that given the large group size, the findings are nevertheless representative of the different syndromes. In the same vein, we also relied entirely on parent-reported anxiety and ER strategy use. While short anxiety scales exist with 10 items (84), eight items (68), and four items (85), to name a few examples, assessment of anxiety with a single item can also be found in the literature (51, 86). Such measures have often been developed in the context of particularly difficult circumstances, such as assessing anxiety in critically ill patients. Given that parents and caregivers of children with SEND experienced a difficult time in the early months of the COVID-19 pandemic [for example Toseeb et al. (47)], it was our goal to keep the survey as short as reasonably possible. We thus opted for a single item to assess anxiety, similar in wording to Turon et al. (51).

Regarding the parent-reported ER strategies, it may be the case that certain ER strategies are less visible to a third party (e.g., a parent or caregiver) leading to under-reporting of that strategy (e.g., *expressive suppression*, perhaps), and may even have led to an over-reporting of the extrinsic ER strategies. This may bias parent and caregiver's responses particularly in individuals that may be less able to communicate about more cognitive, less behavioral strategies which may be more likely in individuals with ID. Nonetheless, parent reports lead to insight about ER in individuals who may not have been able to report in such detail about their own situation, emotional experience, and ER during the first months of the COVID-19 pandemic. As it stands, parent-reported ER questionnaires are not uncommon in the literature (87).

One particular characteristic of this study is that the results are taken from a subsample of responses from participants collected during the first few months of the COVID pandemic. But since we have not collected data before the pandemic, we do not claim that these strategies are either pandemic specific, or that they can be generalized to non-pandemic times. While we assume that similar patterns may be found in other times of individual, family or societal crises, future studies should examine changes in ER strategy use in relation to varying levels of anxiety, for example by using ecological momentary assessment approaches (see above). Since it is likely challenging for parents to know whether a particular ER strategy is used as an attempt to regulate COVID-specific anxieties and stress or other factors not related to the pandemic encountered in daily life, we explicitly asked parents and caregivers to report about ER strategy use in general “these days”, rather than anything COVID-specific. This exploratory study suggests some interesting similarities and differences across groups that future researchers can use to build hypotheses for more structured, less exploratory studies. For example, it would be interesting to extend knowledge about ER strategy choice. Previous research has shown that under highly negative situations, cognitive reappraisal is less effective than distraction (88). It would be interesting to discover if individuals with neurodevelopmental disorders are able to adapt the strategy choice in relation to the negativity of experienced events.

Also, while we focused here on ER strategy *use*, we must emphasize that ER involves other processes that were not considered here, but which would be relevant for further study in neurodevelopmental disorders with and without ID (see above). These processes include the ability to recognize emotions, set regulatory goals, identify and select potential strategies, the ability to implement one or more strategies in combination or in sequence, and other dynamic processes related to ER such as maintaining or disrupting the use of a particular ER strategy in relation to the context and its efficacy (89). In short, the ER field is large, and there is a huge potential for ER studies in SEND, and, more specifically, in neurodevelopmental disorders. Furthermore, although we included a variety of countries (“country” was included as random factor in the analysis), the current study did not focus on the study of cultural or regional differences in the either the caregiver reports or the use of ER strategies. However, other analyses originating from this international collaborative study from which the present data comes will focus on country-specific differences. We hope that the relative strengths of this exploratory study stimulate and further motivate research in this domain, in spite of its relative limitations.

### Conclusions

The present study elucidated different patterns of ER strategy use in different neurodevelopmental disorders. Autistic people without ID were reported to have the largest ER repertoire, including more self-focused cognitive strategies, and were the only group in which we found humor as ER strategy to be linked with lower anxiety levels. For the first time, this study was able to shed light on ER strategy use in individuals with WS, which differed from those of autistic people, which can possibly be accounted for by the well-researched differences in socio-emotional profiles. Moreover, several rather maladaptive strategies were linked to increased anxiety, while *focusing on the positive* was linked to lower anxiety levels across all groups. As such, this exploratory study provides increased insight into ER in WS, ASD with and without ID, and ID-NOS, and highlights the need for further studies on ER strategy use in individuals with neurodevelopmental disorders, using more rigorous assessment tools and that monitor changes of ER strategy use over time. Future studies are required as knowledge about the impact of different ER strategies as potential protective and risk factors on outcomes such as anxiety may also inform interventions to support individuals with neurodevelopmental disorders in their daily lives and in future times of crisis.

## Data availability statement

The raw data supporting the conclusions of this article will be made available by the authors, without undue reservation.

## Ethics statement

The studies involving human participants were reviewed and approved by Ethics Committee of Unidistance Suisse. The patients/participants provided their written informed consent to participate in this study.

## Author contributions

AS, DD, and JV designed and directed the project. NS and AS analyzed the data. AS, NS, DD, and JV wrote the paper. All authors discussed the results, commented on the manuscript, and provided approval for publication of the content.

## Funding

Research Funds of Unidistance Suisse, the European Federation of Williams Syndrome (FEWS) and Swiss National Science Foundation (PP00P1_176722 for AS).

## Conflict of interest

The authors declare that the research was conducted in the absence of any commercial or financial relationships that could be construed as a potential conflict of interest.

## Publisher's note

All claims expressed in this article are solely those of the authors and do not necessarily represent those of their affiliated organizations, or those of the publisher, the editors and the reviewers. Any product that may be evaluated in this article, or claim that may be made by its manufacturer, is not guaranteed or endorsed by the publisher.

## Footnotes

^1^The survey included other questions about other domains, such as a variety of specific worries or ER efficacy, which will be reported elsewhere.

^2^Although we asked not only about parents or caregivers establishing a routine (“I try or my child tries to establish a routine in his/her daily life to lower the experienced stress”), for the purpose of this analysis, we classified this item as extrinsic ER strategy as establishing a routine is likely to involve the parents' consent, advice or help. The survey is fully available here: Van Herwegen et al. (49).

## References

[1] Gross JJ. The emerging field of emotion regulation: an integrative review. Rev Gen Psychol. (1998) 2:271–99. 10.1037/1089-2680.2.3.271

[2] Gross JJ. The extended process model of emotion regulation: elaborations, applications, future directions. Psychol Inq. (2015) 26:130–7. 10.1080/1047840X.2015.989751

[3] Gross JJ Jazaieri H. Emotion, emotion regulation, and psychopathology: An affective science perspective. Clin Psychol Sci. (2014) 2:387–401. 10.1177/2167702614536164

[4] Tajik-Parvinchi D Farmus L Tablon Modica P Cribbie RA Weiss JA. The role of cognitive control and emotion regulation in predicting mental health problems in children with neurodevelopmental disorders. Child Care Health Dev. (2021) 47:608–17. 10.1111/cch.12868 33772823

[5] England-Mason G. Emotion regulation as a transdiagnostic feature in children with neurodevelopmental disorders. Curr Dev Disord Rep. (2020) 7:130–8. 10.1007/s40474-020-00200-2

[6] Mazefsky CA. Emotion regulation and emotional distress in autism spectrum disorder: Foundations and considerations for future research. J Autism Dev Disord. (2015) 45:3405–8. 10.1007/s10803-015-2602-7 26391886 PMC4609632

[7] Cai RY Richdale AL Uljarevi,ć M Dissanayake C Samson AC. Emotion regulation in autism spectrum disorder: where we are and where we need to go. Autism Res. (2018) 11:962–78. 10.1002/aur.1968 29979494

[8] Sáez-Suanes GP García-Villamisar D del Pozo Armentia A Dattilo J. Emotion regulation as a mediator between depressive symptoms and autism spectrum disorder (ASD) in adults with ASD and intellectual disabilities. Res Autism Spectr Disord. (2020) 78:101654. 10.1016/j.rasd.2020.101654

[9] Nozaki Y Mikolajczak M. Extrinsic emotion regulation. Emotion. (2020) 20:10–5. 10.1037/emo0000636 31961171

[10] Strømme P Bjørnstad PG Ramstad K. Prevalence estimation of Williams syndrome. J Child Neurol. (2002) 17:269–71. 10.1177/088307380201700406 12088082

[11] Zeidan J Fombonne E Scorah J Ibrahim A Durkin MS Saxena S. Global prevalence of autism: a systematic review update. Autism Res. (2022) 15:778–90. 10.1002/aur.2696 35238171 PMC9310578

[12] Jones W Bellugi U Lai Z Chiles M Reilly J Lincoln A. Hypersociability in Williams syndrome. J Cogn Neurosci. (2000) 12:30–46. 10.1162/089892900561968 10953232

[13] Riby DM Hancock PJ. Viewing it differently: Social scene perception in Williams syndrome and autism. Neuropsychologia. (2008) 46:2855–60. 10.1016/j.neuropsychologia.2008.05.003 18561959

[14] Riby D Hancock PJ. Looking at movies and cartoons: Eye-tracking evidence from Williams syndrome and autism. J Intellect Disabil Res. (2009) 53:169–81. 10.1111/j.1365-2788.2008.01142.x 19192099

[15] Riby DM Doherty-Sneddon G Bruce V. Atypical unfamiliar face processing in Williams syndrome: what can it tell us about typical familiarity effects? Cogn Neuropsychiatry. (2008) 13:47–58. 10.1080/13546800701779206 18092225

[16] Vivanti G Hocking DR Fanning P Dissanayake C. The social nature of overimitation: Insights from autism and Williams syndrome. Cognition. (2017) 161:10–8. 10.1016/j.cognition.2017.01.008 28088702

[17] Lai PT Ng R Bellugi U. Parents' perspective on the social traits observed in school-age children with autism and children with Williams syndrome. Res. Pract. Intellect. Dev. Disabil. (2021) 2:174–84. 10.1080/23297018.2021.1934893 PMC11168778 38868455

[18] Loveland KA Tunali-Kotoski B. The School-Age Child with an Autistic Spectrum Disorder. In: Volkmar FR Paul R Klin A Cohen D. (Eds.). Handbook of autism and pervasive developmental disorders: Diagnosis, development, neurobiology, behavior. John Wiley and Sons Inc. (2005) p. 247–287. 10.1002/9780470939345.ch9

[19] Asada K Itakura S. Social phenotypes of autism spectrum disorders and Williams syndrome: similarities and differences. Front Psychol. (2012) 3:247. 10.3389/fpsyg.2012.00247 22866045 PMC3408113

[20] Glod M Riby DM Rodgers J. Relationships between sensory processing, repetitive behaviors, anxiety, and intolerance of uncertainty in autism spectrum disorder and Williams syndrome. Autism Res. (2019) 12:759–65. 10.1002/aur.2096 30919599

[21] Klein-Tasman BP Phillips KD Lord CE Mervis CB Gallo F. Overlap with the autism spectrum in young children with Williams syndrome. J Dev Behav Pediatr. (2009) 30:289. 10.1097/DBP.0b013e3181ad1f9a 19668090 PMC2763277

[22] Klein-Tasman BP Li-Barber KT Magargee ET. Honing in on the social phenotype in Williams syndrome using multiple measures and multiple raters. J Autism Dev Disord. (2011) 41:341–51. 10.1007/s10803-010-1060-5 20614173 PMC3020248

[23] Boulton KA Porter MA. Extending the positive bias in Williams syndrome: the influence of biographical information on attention allocation. Dev Psychopathol. (2020) 32:243–56. 10.1017/S0954579418001712 30728089

[24] Sifneos PE. The prevalence of ‘alexithymic' characteristics in psychosomatic patients. Psychother Psychosom. (1973) 22:255–62. 10.1159/000286529 4770536

[25] Kinnaird E Stewart C Tchanturia K. Investigating alexithymia in autism: a systematic review and meta-analysis. European Psychiatry. (2019) 55:80–9. 10.1016/j.eurpsy.2018.09.004 30399531 PMC6331035

[26] Rodgers J Riby DM Janes E Connolly B McConachie H. Anxiety repetitive behaviours in autism spectrum disorders and Williams syndrome: a cross-syndrome comparison. J Autism Dev Disord. (2012) 42:175–80. 10.1007/s10803-011-1225-x 21424863

[27] Jolliffe R Adams D Simpson K. Trait anxiety in individuals on the autism spectrum: a systematic review. Rev J Autism Dev Disord. (2022). 10.1007/s40489-022-00308-8

[28] Van Steensel FJ Bögels SM Perrin S. Anxiety disorders in children and adolescents with autistic spectrum disorders: a meta-analysis. Clin Child Fam Psychol Rev. (2011) 14:302–17. 10.1007/s10567-011-0097-0 21735077 PMC3162631

[29] Leyfer OT Woodruff-Borden J Klein-Tasman BP Fricke JS Mervis CB. Prevalence of psychiatric disorders in 4 to 16-year-olds with Williams syndrome. Am J Med Genet Part B Neuropsychiatr Genet. (2006) 141B:615–22. 10.1002/ajmg.b.30344 16823805 PMC2561212

[30] Royston R Howlin P Waite J Oliver C. Anxiety disorders in Williams syndrome contrasted with intellectual disability and the general population: a systematic review and meta-analysis. J Autism Dev Disord. (2017) 47:3765–77. 10.1007/s10803-016-2909-z 27696186 PMC5676825

[31] Dykens EM. Anxiety, fears and phobias in persons with Williams syndrome. Dev Neuropsychol. (2003) 23:291–316. 10.1207/S15326942DN231&amp;2_13 12730029

[32] Graham JM Rosner B Dykens E Visootsak J. Behavioral features of CHARGE syndrome (Hall-Hittner syndrome) comparison with Down syndrome, Prader-Willi syndrome, Williams syndrome. Am J Med Genet. (2005) 133:240–7. 10.1002/ajmg.a.30543 15637708

[33] Dimitropoulos A Ho A Klaiman C Koenig K Schultz R. A comparison of behavioral and emotional characteristics in children with autism, Prader-Willi syndrome, Williams syndrome. J Ment Health Res Intellect Disabil. (2009) 2:220–43. 10.1080/1931586090305220415637708

[34] South M Hanley M Normansell-Mossa K Russel NCC Cawthorne T Riby DM. “Intolerance of uncertainty” mediates the relationship between social profile and anxiety in both Williams syndrome and autism. Autism Res. (2021) 14:1986–95. 10.1002/aur.2554 34110083

[35] Phillips KD. Emotion regulation and dysregulation in children and adolescents with Williams syndrome (Publication No. 3332181) [Doctoral dissertation]. University of Wisconsin-Milwaukee. ProQuest Dissertations and Theses Global. (2008).

[36] Pitts C Klein-Tasman B Osborne J Mervis C. Predictors of specific phobia in children with Williams syndrome. J Intellect Disabil Res. (2016) 60:1031–42. 10.1111/jir.12327 27545817 PMC5026631

[37] Woodruff-Borden J Kistler DJ Henderson DR Crawford NA Mervis CB. Longitudinal course of anxiety in children and adolescents with Williams syndrome. Am J Med Genet C Semin Med Genet. (2010) 154:277–90. 10.1002/ajmg.c.30259 20425787 PMC2914498

[38] Dubey N Podder P Pandey D. Knowledge of COVID-19 and its influence on mindfulness, cognitive emotion regulation and psychological flexibility in the Indian community. Front Psychol. (2020) 11:589365. 10.3389/fpsyg.2020.589365 33281687 PMC7689361

[39] Ferreira F de O Lopes-Silva JB Siquara GM Manfroi EC De Freitas. PM. Coping in the Covid-19 pandemia: how different resources and strategies can be risk or protective factors to mental health in the Brazilian population. Health Psychol Behav Med. (2021) 9:182–205. 10.1080/21642850.2021.1897595 34104556 PMC8158238

[40] Kim JH Shim Y Choi I Choi E. The role of coping strategies in maintaining well-being during the COVID-19 outbreak in South Korea. Soc Psychol Personal Sci. 1–13. (2021). 10.1177/1948550621990595

[41] Petrocchi S Levante A Bianco F Castelli I Lecciso F. Maternal distress/coping and children's adaptive behaviors during the COVID-19 lockdown: mediation through children's emotional experience. Front Public Health. (2020) 8:587833. 10.3389/fpubh.2020.587833 33330330 PMC7711130

[42] Wang H Xia Q Xiong Z Li Z Xiang W Yuan Y. The psychological distress and coping styles in the early stages of the 2019 coronavirus disease (COVID-19) epidemic in the general mainland Chinese population: A web-based survey. PLOS ONE. (2020) 15:e0233410. 10.1371/journal.pone.0233410 32407409 PMC7224553

[43] Wang QQ Fang YY Huang HL Lv WJ Wang XX Yang TT. Anxiety, depression and cognitive emotion regulation strategies in Chinese nurses during the COVID-19 outbreak. J Nurs Manag. (2021) 29:1263–74. 10.1111/jonm.13265 33480056 PMC8013387

[44] Yan L Gan Y Ding X Wu J Duan H. The relationship between perceived stress and emotional distress during the COVID-19 outbreak: effects of boredom proneness and coping style. J Anxiety Disord. (2021) 77:102328. 10.1016/j.janxdis.2020.102328 33160275 PMC7598556

[45] Di Renzo M Di Castelbianco FB Vanadia E Petrillo M D'Errico S Racinaro L. (2020). Parent-reported behavioural changes in children with autism spectrum disorder during the COVID-19 lockdown in Italy. Continuity in Education. 1:117–25. 10.5334/cie.20 PMC11104382 38774533

[46] Sideropoulos V Dukes D Hanley M Palikara O Rhodes S Riby D. The impact of COVID-19 on anxiety and worries for families of individuals with special education needs and disabilities in the UK. J Autism Dev Disord. (2021) 52:2656–69. 10.31234/osf.io/gyhd9 34196890 PMC8246131

[47] Toseeb U Asbury DK Code A Fox L Deniz E. Supporting families with children with special educational needs disabilities during COVID-19. PsyArXiv. (2020). Available online at: https://psyarxiv.com/tm69k/

[48] Breaux R Dvorsky MR Marsh NP Green CD Cash AR Shroff DM. Prospective impact of COVID-19 on mental health functioning in adolescents with and without ADHD: protective role of emotion regulation abilities. J Child Psychol Psychiatry. (2021) 62:1132–9. 10.1111/jcpp.13382 33543486 PMC8014657

[49] Van Herwegen J Dukes D Samson A. COVID19 Crisis Response Survey for families of Individuals with Special Needs [Data set]. OSFHOME. (2020). Available online at: osf.io/5nkq9 (accessed May 27, 2020).

[50] Dukes D Van Herwegen J Alessandri M Alnemary F Rad JA Lavenex PB. Introducing the COVID-19 crisis special education needs coping survey. PsyArXiv. 1–15. (2021). 10.31234/osf.io/rtswa

[51] Turon H Carey M Boyes A Hobden B Dilworth S Sanson-Fisher R. Agreement between a single-item measure of anxiety and depression and the Hospital Anxiety and Depression Scale: a cross-sectional study. PLoS ONE. (2019) 14: e0210111. 10.1371/journal.pone.0210111 30608969 PMC6319715

[52] Lewis EJ Yoon KL Joormann J. Emotion regulation and biological stress responding: associations with worry, rumination, and reappraisal. Cogn Emot. (2018) 32:1487–98. 10.1080/02699931.2017.1310088 28397544

[53] Gross JJ John OP. Individual differences in two emotion regulation processes: implications for affect, relationships, and well-being. J Pers Soc Psychol. (2003) 85:348–62. 10.1037/0022-3514.85.2.348 12916575

[54] Samson AC Wells WM Phillips JM Hardan AY Gross JJ. Emotion regulation in autism spectrum disorder: Evidence from parent interviews and children's daily diaries. J Child Psychol Psychiatry. (2015) 56:903–13. 10.1111/jcpp.12370 25442191

[55] Quoidbach J Berry EV Hansenne M Mikolajczak M. Positive emotion regulation and well-being: comparing the impact of eight savoring and dampening strategies. Pers Individ Dif. (2010) 49:368–73. 10.1016/j.paid.2010.03.048

[56] Kugler L Kuhbandner C. That's not funny!–But it should be: Effects of humorous emotion regulation on emotional experience and memory. Front Psychol. (2015) 6:1296. 10.3389/fpsyg.2015.01296 26379608 PMC4551820

[57] Samson AC Gross JJ. The dark and light sides of humor: An emotion-regulation perspective. In: Gruber J Moskowitz TJ. (Eds.). Positive emotion: Integrating the light sides and dark sides. Oxford University Press. (2014) p. 169–182. 10.1093/acprof:oso/9780199926725.003.0010

[58] MATLAB. Version 9.5.0 (R2018b). The MathWorks Inc. (2018). Available online at: https://ch.mathworks.com/de/products/matlab.html

[59] R Core Team,. R: A language environment for statistical computing. Vienna, Austria: R Foundation for Statistical Computing. (2020). Available online at: https://www.R-project.org/

[60] Bates D Maechler M Bolker B Walker S. Fitting linear mixed-effects models using lme4. J Stat Softw. (2015) 67:1–48. 10.18637/jss.v067.i01

[61] Kuznetsova A Brockhoff PB Christensen RHB. lmerTest package: tests in linear mixed effects models. J Stat Softw. (2017) 82:1–26. 10.18637/jss.v082.i13

[62] Lenth RV,. Emmeans: Estimated Marginal Means, aka Least-Squares Means. R package version 1.6.2-1. (2021). Available online at: https://cran.r-project.org/web/packages/emmeans/index.html

[63] Fox J Weisberg S. Visualizing fit and lack of fit in complex regression models with predictor effect plots and partial residuals. J Stat Softw. (2018) 87:1–27. 10.18637/jss.v087.i09

[64] Wickham H,. (2016). ggplot2: Elegant Graphics for Data Analysis. New York: Springer-Verlag. Available online at: https://ggplot2.tidyverse.org

[65] Guller B Yaylaci F Eyuboglu D. Those in the shadow of the pandemic: impacts of the COVID-19 outbreak on the mental health of children with neurodevelopmental disorders and their parents. Int J Dev Disabil. (2021) 1–13. 10.1080/20473869.2021.1930827 PMC9788683 36568626

[66] Mutluer T Doenyas C Aslan Genc H. Behavioral implications of the covid-19 process for autism spectrum disorder, and individuals' comprehension of and reactions to the pandemic conditions. Front Psychiatry. (2020) 11. 10.3389/fpsyt.2020.561882 33304279 PMC7701051

[67] Kent R Simonoff E. Prevalence of anxiety in autism spectrum disorders. In: Kerns CM Renno P Storch EA Kendall PC Wood JJ, editors. Anxiety in Children and Adolescents with Autism Spectrum Disorder: Evidence-Based Assessment and Treatment. Academic Press (2017). p. 5–32. 10.1016/B978-0-12-805122-1.00002-8

[68] Reardon T Spence SH Hesse J Shakir A Creswell C. Identifying children with anxiety disorders using brief versions of the Spence Children's Anxiety Scale for children, parents, and teachers. Psychol Assess. (2018) 30:1342–55. 10.1037/pas0000570 29902050 PMC6179143

[69] Davies M Udwin O Howlin P. Adults with Williams syndrome: preliminary study of social, emotional, behavioural difficulties. Br J Psychiatry. (1998) 172:273–6. 10.1192/bjp.172.3.273 9614479

[70] Ng R Järvinen A Bellugi U. Toward a deeper characterization of the social phenotype of Williams syndrome: the association between personality and social drive. Res Dev Disabil. (2014) 35:1838–49. 10.1016/j.ridd.2014.04.015 24794322 PMC4053572

[71] Treichel N Dukes D Barisnikov K Samson AC. How cognitive, social, and emotional profiles impact humor appreciation: Sense of humor in autism spectrum disorder and Williams syndrome. Humor Res. (2022) 35:113–33. 10.1515/humor-2021-0038

[72] Valla JM Belmonte MK. Detail-oriented cognitive style and social communicative deficits, within and beyond the autism spectrum: Independent traits that grow into developmental interdependence. Dev Rev. (2013) 33:371–98. 10.1016/j.dr.2013.08.004

[73] Gotham K Bishop SL Hus V Huerta M Lund S Buja A. Exploring the relationship between anxiety and insistence on sameness in autism spectrum disorders. Autism Res. (2013) 6:33–41. 10.1002/aur.1263 23258569 PMC4373663

[74] McClure KS Halpern J Wolper PA Donahue JJ. Emotion regulation and intellectual disability. J Develop Disabil. (2009) 15:38–44.

[75] Benson BA Fuchs C. Anger-arousing situations and coping responses of aggressive adults with intellectual disability. J Intellect Develop Disabil. (1999) 24:207–14. 10.1080/13668259900033991

[76] te Brinke LW Schuiringa HD Matthys W. Emotion regulation and angry mood among adolescents with externalizing problems and intellectual disabilities. Res Develop Disabil. (2021) 109:103833. 10.1016/j.ridd.2020.103833 33421677

[77] Sheppes G Gross JJ. Is timing everything? Temporal considerations in emotion regulation. Pers Soc Psychol Rev. (2011) 15:319–31. 10.1177/1088868310395778 21233326

[78] Stinson L Liu Dallery J. Ecological momentary assessment: A systematic review of validity research. Pers Behav Sci. (2022) 45:469–93. 10.1007/s40614-022-00339-w 35719870 PMC9163273

[79] Klein-Tasman BP Young BN Levine K Rivera K Miecielica EJ Yund BD. Acceptability and effectiveness of humor- and play-infused exposure therapy for fears in Williams syndrome. Evidence-based practice in child and adolescent. MentHealth. (2022) 1:94–111. 10.1080/23794925.2021.1950080

[80] Zaharia A Noir-Kahlo K Bressoud N Sander D Dukes D Samson AC. Proof of concept: a brief psycho-educational training program to increase the use of positive emotion regulation strategies in individuals with autism spectrum disorder. Front Psychol. (2021) 12:705937. 10.3389/fpsyg.2021.705937 34790142 PMC8591291

[81] Samson AC Hegenloh M. Stimulus properties affect humor processing in individuals with Asperger syndrome. J Autism Dev Disord. (2010) 40:438–47. 10.1007/s10803-009-0885-2 19859795

[82] Samson AC. Humor(lessness) elucidated - sense of humor in individuals with Autism Spectrum Disorders: Review and introduction. Int J Humor Res. (2013) 26:393–409. 10.1515/humor-2013-0027

[83] Lord C Rutter M DiLavore P Risi S Gotham K Bishop S. Autism diagnostic observation schedule−2nd edition (ADOS-2). Los Angeles, CA: Western Psychological Corporation. (2012).

[84] Bergua V Meillon C Potvin O Ritchie K Tzourio C Bouisson J. Short STAI-Y anxiety scales: validation and normative data for elderly subjects. Aging Ment health. (2016) 20:987–95. 10.1080/13607863.2015.1051511 26055726

[85] Kroenke K Spitzer RL Williams JBW Löwe B. An ultra-brief screening scale for anxiety and depression: the PHQ–4. Psychosomatics. (2009) 50: 613–21. 10.1016/S0033-3182(09)70864-3 19996233

[86] McKinley S Coote K Stein-Parbury J. Development testing of a Faces Scale for the assessment of anxiety in critically ill patients. J Adv Nurs. (2003) 41:73–9. 10.1046/j.1365-2648.2003.02508.x 12519290

[87] Gullone E Hughes EK King NJ Tonge B. The normative development of emotion regulation strategy use in children and adolescents: a 2-year follow-up study. J Child Psychol Psychiatry. (2010) 51:567–74. 10.1111/j.1469-7610.2009.02183.x 19845818

[88] Sheppes G Scheibe S Suri G Gross JJ. Emotion regulation choice. Psychological Sci. (2011) 22:1391–6. 10.1177/0956797611418350 21960251

[89] Cai RY Samson AC. Emotion regulation in individuals on the autism spectrum. In: Gross JJ Ford B. (Eds). Handbook of Emotion Regulation. [Manuscript accepted for publication]. (2022).

