# Supplementary material for: An exploratory study on emotion regulation strategy use in individuals with Williams syndrome, autism spectrum disorder and intellectual disability
Source: Front Psychiatry. 2022 Nov 23;13:940872. doi: 10.3389/fpsyt.2022.940872 (PMC9726902; doi:10.3389/fpsyt.2022.940872)
Supplement: Supplementary file 1 [file Data_Sheet_1.docx]

**Supplementary Material**

1. **Country Residency**

**Table A1**

*Frequency of residency in the countries ordered from highest to lowest*

| Country | Frequency per country | | |
| --- | --- | --- | --- |
| Saudi Arabia | | 762 |  |
| United States of America | | 256 |  |
| Germany | | 252 |  |
| China | | 171 |  |
| United Kingdom | | 153 |  |
| Switzerland | | 137 |  |
| Russia | | 68 |  |
| Portugal | | 56 |  |
| Australia | | 55 |  |
| Italy, Sweden | | 41 |  |
| Czech Republic | | 30 |  |
| Ireland | | 29 |  |
| France and Iran | | 26 |  |
| Romania and Slowakia | | 23 |  |
| Spain | | 21 |  |
| Ecuador | | 19 |  |
| Austria | | 17 |  |
| Brazil | | 13 |  |
| Canada | | 9 |  |
| Greece and India | | 6 |  |
| Mexico | | 5 |  |
| Belarus, Netherlands and Peru | | 4 |  |
| Belgium and Trinidad and Tobago | | 3 |  |
| Egypt, Isreal, New Zealand and Singapore | | 2 |  |
| Argentina, Bahrain, Bolivia, Central African Republic, Chile, Cyprus, Democratic Republic Korea, Denmark, Jordan, Kazakhstan, Lithuania, Luxembourg, Namibia, Norway, Slovenia, South Africa and United Arab Emirates | | 1 |  |

*Note*. *N* = 2288.

**B. Emotion regulation instructions and items**

**Instructions and items to assess emotion regulation strategy use (frequency; see also Van Herwegen et al., 2020)**

We would like to know the emotion regulation strategies that your child with SEND uses to cope these days. For example, in order to feel less stressed. How successful are his/her strategies? And what are you doing to help him/her?

In the following questions we are asking for the **FREQUENCY**, i.e. how often is your child using a certain strategy on a scale of **1 = very rarely** **to 5 = very frequently.**

1. [Information avoidance:] In order to feel less stressed, my child avoids any information about it (e.g., COVID-19).

2. [Information search:] In order to feel less stressed, my child gets as much information as possible.

3. [Sharing/Talking about:] In order to feel less stressed, my child talks about it as often as possible.

4. [Distraction:] In order to feel less stressed, my child distracts him or herself as much as possible.

5. [Cognitive reappraisal:] In order to feel less stressed, my child changes the way he or she is thinking about the situation.

6. [Focusing on the positive:] In order to feel less stressed, my child focuses on positive aspects/ views the situation in a different light (e.g., more family time together).

7. [Humor:] In order to feel less stressed, my child tells jokes and engages in humor.

8. [Expressive suppression:] In order to feel less stressed, my child does not express negative emotions (i.e., suppression of emotions).

9. [Rumination:] In order to feel less stressed, my child ruminates (i.e., thinks deeply about something).

10. [Aggressive behaviors:] In order to feel less stressed, my child engages in aggressive behaviors towards others around him/her.

11. [Isolation/withdrawal:] In order to feel less stressed, my child isolates himself/herself in his/her room, or another room of the house.

12. [Repetitive behaviors:] In order to feel less stressed, my child engages in repetitive behaviors (asking the same questions repetitively, repeatedly washing their hands, rocking or other stereotypic behaviors (stimming), etc.).

13. [Parent shielding:] I try to shield my child from the situation as much as possible.

14. [Parent routine:] I try or my child tries to establish a routine in his/her daily life to lower the experienced stress.

*Note: The [labels] of the strategies were not presented in the questionnaire. The order of the items is presented as in the survey, not as they are discussed in the paper.*

1. **Emotion regulation frequency per group**

**Table C1**
*This table includes the average use of each emotion regulation strategy (frequency) and confidence interval for all 4 groups*

|  | ASD without ID | | | ASD with ID | | | ID-NOS | | | WS | |  |
| --- | --- | --- | --- | --- | --- | --- | --- | --- | --- | --- | --- | --- |
| ER strategies | *M* | 95% CI | *M* | | 95% CI | *M* | | 95% CI | *M* | | 95% CI | |
| Isolation/withdrawal | 2.70 | [2.57, 2.83] | 2.23 | | [2.13, 2.33] | 1.86 | | [1.76, 1.96] | 2.00 | | [1.85, 2.14] | |
| Information avoidance | 1.97 | [1.87, 2.07] | 1.79 | | [1.70, 1.88] | 1.83 | | [1.73, 1.92] | 1.78 | | [1.64, 1.92] | |
| Information search | 2.12 | [2.00, 2.23] | 1.73 | | [1.64, 1.82] | 1.79 | | [1.69, 1.88] | 2.03 | | [1.86, 2.19] | |
| Rumination | 2.42 | [2.30, 2.53] | 1.91 | | [1.82, 2.00] | 1.81 | | [1.72, 1.90] | 2.11 | | [1.95, 2.28] | |
| Expressive suppression | 1.88 | [1.78, 1.98] | 1.77 | | [1.68, 1.85] | 1.91 | | [1.81, 2.00] | 1.68 | | [1.55, 1.82] | |
| Aggressive behaviors | 2.20 | [2.10, 2.31] | 2.10 | | [2.00, 2.19] | 1.97 | | [1.87, 2.07] | 1.77 | | [1.62, 1.91] | |
| Repetitive behaviors | 2.64 | [2.52, 2.77] | 2.79 | | [2.67, 2.90] | 2.22 | | [2.10, 2.34] | 2.66 | | [2.46, 2.84] | |
| Sharing/talking about COVID-19 | 2.03 | [1.91, 2.13] | 1.76 | | [1.67, 1.84] | 1.85 | | [1.75, 1.94] | 2.29 | | [2.12, 2.46] | |
| Distraction | 2.65 | [2.52, 2.77] | 2.09 | | [1.89, 2.18] | 1.97 | | [1.87, 2.07] | 2.42 | | [2.25, 2.59] | |
| Cognitive reappraisal | 1.64 | [1.55, 1.72] | 1.67 | | [1.58, 1.74] | 1.69 | | [1.60, 1.78] | 1.65 | | [1.53, 1.77] | |
| Focusing on the positive | 2.08 | [1.97, 2.19] | 1.84 | | [1.75, 1.92] | 1.95 | | [1.84, 2.05] | 2.25 | | [2.08, 2.41] | |
| Humor | 2.00 | [1.89, 2.11] | 1.61 | | [1.53, 1.68] | 1.83 | | [1.73, 1.92] | 2.00 | | [1.84, 2.15] | |
| Parent shielding | 2.71 | [2.58, 2.84] | 2.62 | | [2.51, 2.73] | 2.35 | | [2.23, 2.46] | 2.96 | | [2.78, 3.13] | |
| Parent routine | 3.32 | [3.20, 3.44] | 2.92 | | [2.81, 3.03] | 2.42 | | [2.30, 2.53] | 3.22 | | [3.04, 3.39] | |

*Note*. CI = confidence interval; ER = emotion regulation; ASD = autism spectrum disorder; ID = intellectual disability; ID-NOS = intellectual disability not otherwise specified; WS = Williams syndrome.

1. **Pairwise differences between groups for isolation/withdrawal**

**Table D1**

This table includes the t and p values that correspond to the pairwise differences between groups for the ER strategy of isolation/withdrawal

| Pairwise differences | *t* | *p* |
| --- | --- | --- |
| ASD without ID vs ASD with ID | 4.91 | <.001 |
| ASD without ID vs ID-NOS | 8.02 | <.001 |
| ASD without ID vs WS | 8.72 | <.001 |
| ASD with ID vs ID-NOS | 4.40 | <.01 |
| ASD with ID vs WS | 5.39 | <.001 |
| ID-NOS vs WS | 1.96 | *>.05* |

*Note*. ASD = autism spectrum disorder; ID = intellectual disability; ID-NOS = intellectual disability not otherwise specified; WS = Williams syndrome.

1. **Further information about group differences in the link between emotion regulation and anxiety**

We found significant effects of age; *F*(1,2172)=18.56, *p*<.001, and of the same seven ER strategies (Information avoidance; *F*(1, 2160) = 3.96, *p* < .05, Information search; *F*(1, 2168) = 7.32, *p*  < .01, Distraction; *F*(1, 2168) = 22.51, *p* < .001, Rumination; *F*(1, 2167) = 40.85, *p* < .001, Aggressive behaviors; *F*(1, 2168) = 10.83, *p* < .001, Repetitive behaviors; *F*(1, 2169) = 16.68, *p* < .001, Parent shielding; *F*(1, 2099) = 21.81, *p* < .001, Focusing on the positive; *F*(1, 2171) = 11.18, *p* < .001) as in section 3.4, but not for gender; *F*(1,2167)=0.71, *p*>.05, or group; *F*(3,2167)=0.7, *p*>.05.
